# Honeybee (*Apis mellifera*)-associated bacterial community affected by American foulbrood: detection of *Paenibacillus larvae* via microbiome analysis

**DOI:** 10.1038/s41598-017-05076-8

**Published:** 2017-07-11

**Authors:** Tomas Erban, Ondrej Ledvinka, Martin Kamler, Marta Nesvorna, Bronislava Hortova, Jan Tyl, Dalibor Titera, Martin Markovic, Jan Hubert

**Affiliations:** 1 Crop Research Institute, Drnovska 507/73, Prague 6-Ruzyne, CZ-161 06 Czechia; 2 Czech Hydrometeorological Institute, Na Sabatce 2050/17, Prague 412, CZ-143 06 Czechia; 3 Bee Research Institute at Dol, Maslovice-Dol 94, Libcice nad Vltavou, CZ-252 66 Czechia; 4 Department of Zoology and Fisheries, Faculty of Agrobiology, Food and Natural Resources, Czech University of Life Sciences Prague, Prague 6-Suchdol, Czechia

## Abstract

Honeybee (*Apis mellifera* L.) workers act as passive vectors of *Paenibacillus larvae* spores, which cause the quarantine disease American foulbrood (AFB). We assessed the relative proportions of *P. larvae* within the honeybee microbiome using metabarcoding analysis of the 16 S rRNA gene. The microbiome was analyzed in workers outside of the AFB zone (control - AFB0), in workers from asymptomatic colonies in an AFB apiary (AFB1), and in workers from colonies exhibiting clinical AFB symptoms (AFB2). The microbiome was processed for the entire community and for a cut-off microbiome comprising pathogenic/environmental bacteria following the removal of core bacterial sequences; varroosis levels were considered in the statistical analysis. No correlation was observed between AFB status and varroosis level, but AFB influenced the worker bee bacterial community, primarily the pathogenic/environmental bacteria. There was no significant difference in the relative abundance of *P. larvae* between the AFB1 and AFB0 colonies, but we did observe a 9-fold increase in *P. larvae* abundance in AFB2 relative to the abundance in AFB1. The relative sequence numbers of *Citrobacter freundii* and *Hafnia alvei* were higher in AFB2 and AFB1 than in AFB0, whereas *Enterococcus faecalis*, *Klebsiella oxytoca*, *Spiroplasma melliferum* and *Morganella morganii* were more abundant in AFB0 and AFB1 than in AFB2.

## Introduction

American foulbrood (AFB) is a quarantine disease of the larvae and pupae of the honeybee, *Apis mellifera* L., and it is listed in the Terrestrial Animal Health Code by the Office International des Epizooties (OIE) of the World Organization for Animal Health^1^. The disease is caused by the gram-positive, spore-forming facultative anaerobic bacterium *Paenibacillus larvae* ^2^, originally described as *Bacillus larvae* ^3^, and it causes substantial economic losses to beekeepers^4^. The spores of *P. larvae* are extremely infectious, but colonies differ in their resistance to AFB outbreaks^5^. Honeybee workers are suitable for the early detection of AFB^6–8^. In a study investigating three honeybee pathogens in Spain, the detected *P. larvae* (as well as *Melissococcus plutonius*) prevalence was two-fold greater in adult bees than in brood samples^8^. Similar *P. larvae* spore loads are found in honeybees isolated from different parts of the colony^6, 7^, indicating homogeneity across different stages of the adult bee life cycle. However, Gillard *et al*.^7^ noted that bees collected at the hive entrance are of limited value for AFB diagnostics^7^. Thus, greater differences in spore loads occur at the level of individual workers^9^, and there is a correlation between the proportion of clinically diseased cells and number of infected workers^9^. Additional knowledge regarding *P. larvae* transmission methods and occurrence in honeybee colonies is required to better understand AFB, which notably contributes to the loss of colonies.

In recent years, numerous techniques have been developed to detect the economically important pathogen *P. larvae*. As noted in the review by de Graaf *et al*.^10^, these microbiological and molecular methods are applicable to brood samples, food reserves (honey, pollen and royal jelly), adult workers, and wax debris (de Graaf *et al*. 2010). The spores of *P. larvae* have been recovered from beeswax^11^ and detected in commercial pollen^12^. However, the list of reviewed techniques did not include high-throughput sequencing (HTS), which has become important for the study of honeybee and bumblebee microbiomes^13–16^. HTS has also been utilized to study interactions between parasites and honeybee-associated bacteria^17, 18^. Currently, HTS is being used to investigate the bacterial community of honeybees in relation to European foulbrood^19^.

The microbiome of mature honeybee workers roughly consists of approximately 10^9^ bacterial cells^13^, and the composition of the bacterial community is likely influenced by the pathogen load in a colony. In the case of *P. larvae*, even a tolerant honeybee colony typically does not exceed 2.5 × 10^5^ spores per g of honey^5^. If such a concentration of spores is present in hive food stores, sequencing analysis of the adults feeding on the contaminated honey or using it to feed the brood will be successful, and moreover, the total microbiome will be influenced by the number of pathogen sequences. In this study, HTS was employed to compare the relative proportions of *P. larvae* in the microbiomes of honeybee workers obtained from colonies with and without clinical symptoms as well as control samples from outside of the AFB zone. *Varroa destructor* infestation was also investigated as a factor in our statistical analyses. Additionally, we performed an AFB microbiome analysis of pupae. The presence of pathogenic *P. larvae* detected in commonly investigated sample types is demonstrated from a novel perspective in this study and the impact of *P. larvae* on the whole microbial community structure is also assessed.

## Results

Overall, the identified bacterial taxa formed 116 and 114 operational taxonomic units (OTUs) at 97% similarity in the worker (Table S1) and pupae (Table S2) samples, respectively. After omitting the pupae samples, the dataset for the total worker honeybee microbiome contained 1,222,779 sequences, ranging from 14,741 to 68,869 sequences per sample. The dataset of the environmental and parasitic bacteria (cut-off microbiome) comprised 78,387 sequences, which formed 92 OTUs, and the sequence numbers ranged from 104 to 16,381 per sample.

### Differences in the worker honeybee microbiome by AFB category

**Figure 1:**
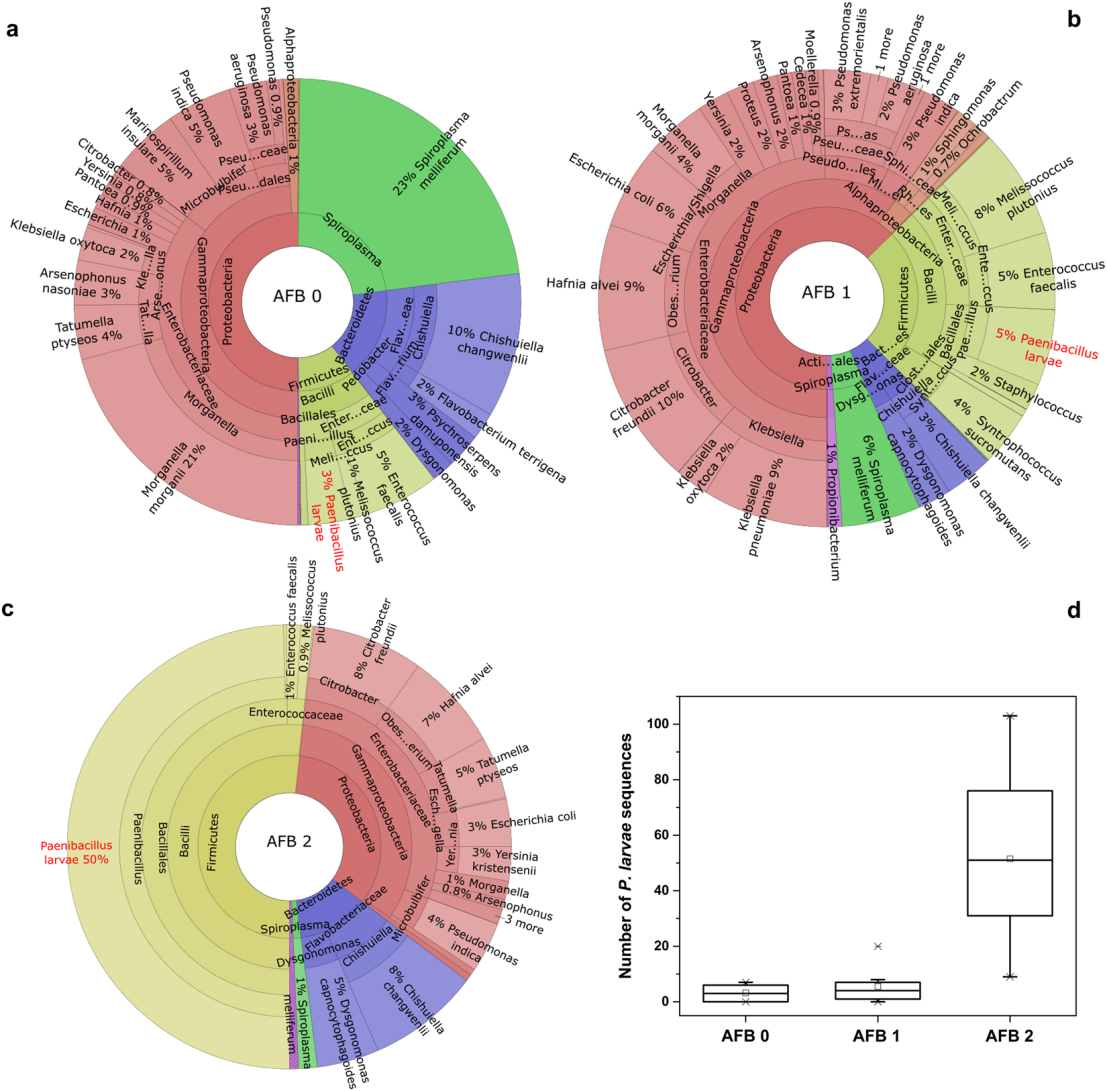
Krona projections of the microbiome of honeybee workers based on analyses of the parasitic/environmental portion of the microbiome and comparison of *P. larvae* sequence numbers. *Paenibacillus larvae* is shown in red. Legend: (**a**) AFB0: control bees from outside the AFB zone with no signs of AFB, (**b**) AFB1: bees from asymptomatic colonies in AFB apiaries, (**c**) AFB2: bees from colonies exhibiting clinical symptoms of AFB, (**d**) comparison of the number of *P. larvae* sequences.

Krona projections (Fig. 1a–c) show differences in the cut-off microbiome structure among AFB0, AFB1 and AFB2; according to these illustrations, *P. larvae* comprised 3% in AFB0, 5% in AFB1 and 50% in AFB2. Although the number of *P. larvae* sequences differed little between AFB0 and AFB1 (Fig. 1d), the Krona projections indicate substantial differences in the cut-off microbiome structure. Figure 1d shows the number of *P. larvae* sequences in AFB0, AFB1 and AFB2; however, the statistical comparison was performed using the relative abundance of *P. larvae* (see OTU3 in Table 1).

**Table 1:** METASTATS analysis results of the relative abundance of the environmental/pathogenic bacteria (cut-off microbiome). Samples of honeybee colonies with (AFB2) and without (AFB1) clinical symptoms from AFB apiaries and control (AFB0) samples outside the AFB zone were compared. Statistical differences are indicated by letters and marked in gray. Significant differences are indicated by P-values (α < 0.05) and are marked in bold.

OTU id	Bacterial taxa (GenBank ID)	aOTU	AFB2	AFB1	AFB0	P-value		
			mean	mean	mean	AFB2/AFB1	AFB2/AFB0	AFB1/AFB0
OTU3	*Paenibacillus larvae*	40,092	0.495 ± 0.092a	0.053 ± 0.020b	0.031 ± 0.009b	**0.000**	**0.000**	0.363
OTU28	*Citrobacter freundii*	3,781	0.081 ± 0.031a	0.100 ± 0.052a	0.007 ± 0.007b	0.781	**0.037**	**0.044**
OTU16	*Spiroplasma melliferum*	3,693	0.013 ± 0.010b	0.056 ± 0.032ab	0.223 ± 0.078a	0.279	**0.005**	0.061
OTU18	*Morganella morganii*	2,866	0.013 ± 0.009b	0.038 ± 0.025ab	0.202 ± 0.102a	0.335	**0.021**	0.106
OTU40	*Hafnia alvei*	2,800	0.070 ± 0.032a	0.089 ± 0.044a	0.011 ± 0.006b	0.714	0.090	**0.050**
OTU49	*Klebsiella pneumoniae*	2,519	0.004 ± 0.002b	0.089 ± 0.041a	0.002 ± 0.001b	**0.013**	0.690	**0.006**
OTU11	*Enterococcus faecalis*	1,049	0.011 ± 0.005b	0.054 ± 0.018a	0.053 ± 0.015a	**0.032**	**0.009**	0.935
OTU52	*Melissococcus plutonius*	614	0.009 ± 0.004b	0.077 ± 0.038a	0.011 ± 0.008b	**0.014**	0.877	**0.050**
OTU128	*Klebsiella oxytoca*	400	0.002 ± 0.001b	0.024 ± 0.010a	0.024 ± 0.020a	**0.029**	0.219	0.969

**Figure 2:**
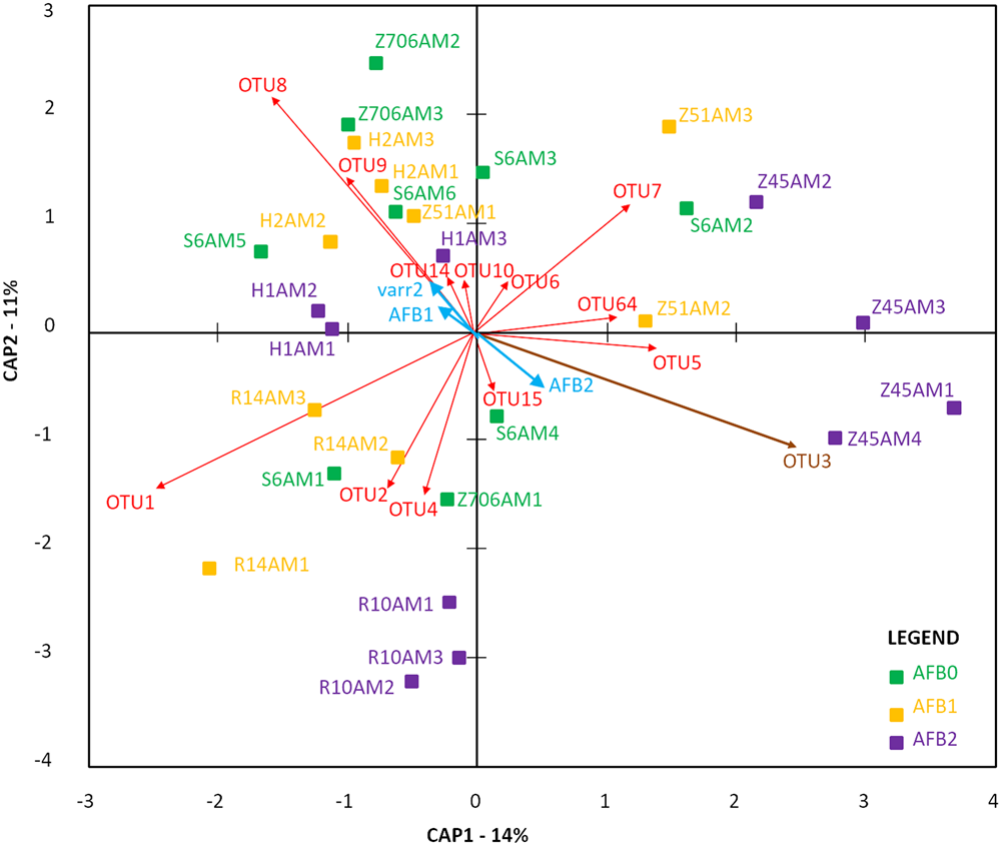
Triplot visualization of principal coordinates in the RDA of the *Apis mellifera* worker microbiome. The analysis was based on the total microbiome dataset and included varroosis and AFB environmental factors. A correlation triplot containing sample scores given by the weighted sums of OTUs was constructed. The first two axes explained 25% of the total variability in the dataset as indicated by the bracketed percentage.

**Figure 3:**
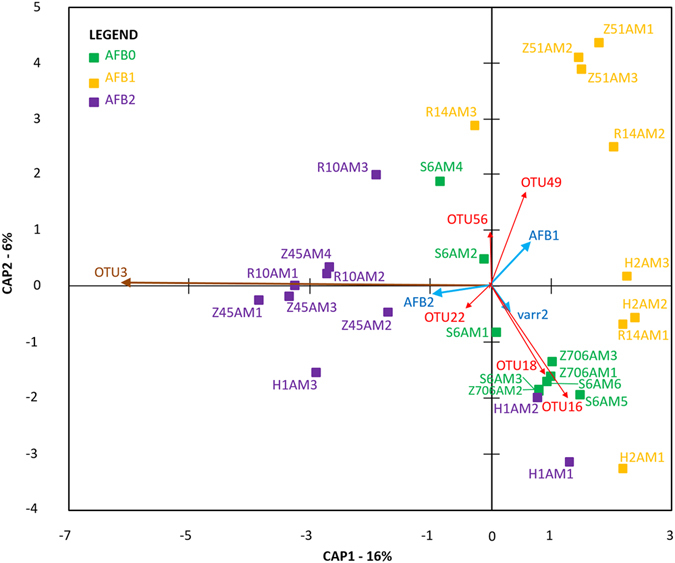
Triplot visualization of principal coordinates in the RDA of the *A. mellifera* worker microbiome. The analysis was based on the parasitic/environmental microbiome dataset and included varroosis and AFB environmental factors. A correlation triplot containing sample scores given by the weighted sums of OTUs was constructed. The first two axes explained 22% of the total variability in the dataset as indicated by the bracketed percentage.

As a factor, AFB was found to have no or only a marginal effect on the number of OTUs in the total parasitic/environmental microbiome datasets; the inverse Simpson diversity index was not affected (Table 2). Redundancy (RDA) analyses, particularly the variance inflation factors (VIFs; Table S3), which sometimes exceeded 1,000, suggested a strong multicollinearity among the environmental variables. Upon further investigation, this multicollinearity was attributable to geographic coordinates, dominated by longitude. Varroosis levels could be added to the models alongside AFB levels. Therefore, we employed partial db-RDA models in which only the coordinates and bee sample collection times were conditioned. When we evaluated all of the OTUs and the pathogenic OTUs separately, varroosis was only important for the former dataset at P < 0.001. Both RDA models were significant (F = 2.82, P < 0.001 and F = 2.36, P < 0.001), which was primarily attributable to the effect of AFB. Interestingly, unlike the case for all OTUs (for which the first two axes were significant; P < 0.01), only the first RDA axis was significant for pathogenic OTUs (P < 0.001). In particular, the triplot visualization for all OTUs suggested a positive correlation between *P. larvae* (OTU3) and the colonies exhibiting clinical signs of AFB (AFB2) (Fig. 2), whereas the core bacteria *Bifidobacterium asteroides* (OTU8) and *Lactobacillus mellis* (OTU9), were negatively correlated with *P. larvae* (OTU3). Additionally, level 2 varroosis (varr2) was closely associated with AFB1 (Fig. 2). Upon constraining the analysis to pathogenic OTUs, the triplot pattern changed, although *P. larvae* (OTU3) remained correlated with AFB2. As shown in Fig. 3, the direction of the varroosis level 2 vector (varr2) differed from that of the AFB1 vector. The OTUs that were positively correlated with varroosis level were *Spiroplasma melliferum* (OTU16) and *Morganella morganii* (OTU18), whereas *Klebsiella pneumoniae* (OTU49) was correlated with AFB1. As a factor, AFB did not influence the distribution of bacteria in the worker microbiome (total microbiome) according to the analysis of molecular variance (AMOVA; Fs = 1.291, P = 0.195), and the sample variability was not significantly different based on the homogeneity of molecular variance test (HOMOVA; Bv = 0.460, P = 0.093). In contrast, the dataset for environmental and parasitic bacteria was significantly influenced by AFB according to the AMOVA (Fs = 4.466, P < 0.001). The Bonferroni-corrected values revealed significant differences (P ≤ 0.05) among all AFB-derived samples. There were no differences in sample variability according to the HOMOVA test (Bv = 0.569, P = 0.311).

**Table 2:** Comparison of alpha-diversity parameters in the honeybee microbiome for both the total microbiome and the microbiome formed by environmental/pathogenic bacteria (cut-off microbiome). Samples from AFB apiaries with (AFB2) and without (AFB1) clinical symptoms and control (AFB0) samples outside the AFB zone were compared.

Microbiome characteristics	Alpha-diversity parameters	AFB0	AFB1	AFB2	Kruskal-Wallis	
		mean ± stdev	mean ± stdev	mean ± stdev	K	P
Total microbiome	nseq	36,561 ± 17,011a	46,646 ± 15,119a	47,192 ± 16,792a	3.174	0.211
	sobs	44,889 ± 4,372a	47 ± 5ab	51 ± 5b	**6.723**	**0.030**
	invsimpson	7,812 ± 1,907a	9 ± 1a	8 ± 1a	2.687	0.267
Cut-off microbiome	nseq	1,246 ± 1,308a	1,106 ± 1,238a	5,722 ± 5,216b	**13.640**	**0.000**
	sobs	23 ± 5a	25 ± 4a	29 ± 5a	**5.918**	**0.047**
	invsimpson	4 ± 3a	7 ± 2a	3 ± 3a	1.043	0.602

Legend: nseq- total number of sequences; sobs – number of OTUs; invsimpson – inverse Simpson diversity index.

METASTATS analyses confirmed the significant influence of AFB on the relative number of *P. larvae* (see OTU3 in Table 1) in the pathogenic/environmental dataset. Higher relative abundances of *P. larvae* were found in AFB2 colonies than in AFB1 and AFB0 colonies; there was no difference between AFB1 and AFB0. However, the AFB1 colonies did not differ significantly from control apiaries outside the zone (AFB0) in the relative number of *P. larvae* (see OTU3 in Table 1). The bacterial taxa with significantly higher relative abundances in AFB2 and AFB1 than in AFB0 were *Citrobacter freundii* (OTU28) and *Hafnia alvei* (OTU40). *Enterococcus faecalis* (OTU11), *Klebsiella oxytoca* (OTU128), *Morganella morganii* (OTU18) and *Spiroplasma melliferum* (OTU16) had higher relative abundances in AFB0 and AFB1 than in AFB2. Finally, *K. pneumoniae* (OTU49) and *Melissococcus plutonius* (OTU52) were present in greater numbers in AFB1 than in AFB0 and AFB2.

### Honeybee pupae

The taxonomic compositions of pupae were visualized in the KRONA projections (see Figure S1), which show the proportions of bacteria in the investigated samples. The bacterial community of the pupae obtained from AFB2 was primarily formed by one eudominant OTU (Table S2). *Paenibacillus larvae* (OTU3) was present in the pupae of AFB2, whereas the following taxa were detected in AFB1 pupae: core bacteria *Lactobacillus apis* (OTU2), *Gilliamella apicola* (OTU5), *Snodgrasella alvi* (OTU7), and *Frischella perrara* (OTU15). *Enterococcus faecalis* (OTU11) was prevalent in the sampled pupae from the control (AFB0) honeybee colonies.

## Discussion

In this study, we analyzed the occurrence of *P. larvae* in AFB-affected apiaries, including colonies exhibiting clinical signs, and in asymptomatic colonies, by employing a HTS approach and comparing the obtained data with those from control samples. Our samples consisted of worker honeybees, which are suitable for AFB diagnostics^6, 20, 21^ and facilitate both horizontal and vertical bacterial transmission^22^. Based on our results, in addition to commonly used microbiological techniques and molecular methods, such as PCR, qPCR and potential proteomics approaches^10^, HTS is useful for detection of *P. larvae* in honeybee samples. The great advantage of HTS is its ability to identify not only the bacterial pathogen *P. larvae* but also other bacteria, including both symbiotic and pathogenic/environmental bacteria, enabling us to demonstrate that AFB can influence the honeybee worker microbiome composition. The results of this study provide insight into the transmission of *P. larvae* and AFB disease development at the microbiome level. Due to the presence of *P. larvae* in control samples, we believe that *P. larvae* survives in honeybee colonies in an enzootic state^23^. Additionally, we visualized differences in the microbiome of pupae in terms of AFB occurrence in colonies using KRONA projections.

### Explanation of *P. larvae* quantitation

The number of spores per adult honeybee depends on the presence or absence of clinical symptoms^21^. According to Gende *et al*.^21^, approximately 3,000 *P. larvae* spores per adult bee may represent the threshold for the appearance of clinical AFB symptoms, and the same authors observed at least a 2-fold reduction in the number of spores in bees from colonies without clinical symptoms relative to the number in bees from colonies with AFB symptoms^21^. Our investigation of the worker honeybee microbiome using HTS revealed approximately 9-fold greater numbers of *P. larvae* in workers from colonies with clinical symptoms compared with the numbers in workers from colonies that were asymptomatic but located at an AFB-diseased site (OTU3 in Table 1). In contrast, there was only a slight, statistically non-significant increase in *P. larvae* abundance in asymptomatic colonies compared with the abundance in colonies used as controls outside the protective zone. Differences in the proportion of *P. larvae* within the microbiome correlated well with KRONA projections depicting the microbiome composition after the removal of symbiotic bacteria (Fig. 1a–c). In summary, following the extraction of parasitic/environmental bacteria, the worker bee microbiome was notably influenced by *P. larvae* in bee colonies that exhibited clinical signs of AFB. These results are important for the interpretation and presentation of HTS data for pathogens of relatively low abundance, and this situation is specific to cases in which the pathogenic agent does not cause disease and is only transmitted by the host. Specifically, worker bees act only as vectors because *P. larvae* do not germinate in the adult gut^24^. Honeybee workers are known to drift between colonies, but they also rob weak and collapsing colonies^25, 26^. Robbing honey contaminated with *P. larvae* spores is an important factor in AFB transmission; larvae in the thief colony can be infected by feeding on stolen honey^5, 27^. According to Gillard *et al*.^7^, who utilized cultivation techniques, spores were detectable in relatively high numbers (greater than 25%) in asymptomatic colonies located in AFB apiaries^7^. Because some spores remain active in the honeybee gut for more than 2 months, honeybees are able to spread bacteria to other members of a colony over a long period of time, allowing increased infection to occur^5^. Thus, even a slight increase in the number of spores in a colony, which we observed for AFB-asymptomatic colonies, is critical for AFB development. However, AFB development is related to pathogen resistance levels in a particular colony; thus, in some colonies, more spores are needed to initiate the clinical signs of AFB^5^.

### The presence of *P. larvae* in control samples indicates an enzootic state

One of our important findings is the presence of low numbers of sequences in control samples obtained outside the AFB zone. This result is supported by HTS analyses in other experiments (unpublished data) unrelated to AFB; in some colonies, we identified reads corresponding to *P. larvae*, whereas in others, no reads corresponding to *P. larvae* were identified. The enzootic occurrence of *P. larvae* has previously been suggested^23^. The recent analysis of a large spectrum of molecular markers suggested that certain endemic populations of *P. larvae* may adapt to the local honeybee population^28^. In this context, there is risk for AFB development due to the exchange of genetic honeybee material among colonies of unknown origin. According to Hansen and Rasmussen^29^, 11% of honey is contaminated with *P. larvae*, and importantly, of the honey samples investigated in their study, 9% were obtained from colonies lacking any signs of AFB symptoms in the same year or the following year^29^. A study examining the prevalence of three pathogens in Spanish apiaries using multiplex PCR detected *P. larvae* in 1.5 to 4.2% of transverse study (samples differing between seasons and over 2 years)^8^. These results reveal the spatial enzootic occurrence of the pathogen, which can occasionally overcome the resilience of colonies and result in outbreaks. Apicultural practices are key for controlling the spread of pathogens such as *P. larvae*, with consideration for possible swarming^22^. An experienced beekeeper is able recognize honeybee colonies that are threatened and prevent future damage stemming from the weakened colony, which is more susceptible to disease, including infection by *P. larvae*. Moreover, an experienced beekeeper can prevent swarming, which represents a risk for uncontrolled pathogen spread. Swarms of unknown origin are particularly dangerous in areas with high concentrations of honeybee colonies.

### The influence of *P. larvae* on other bacteria and the lack of an association between AFB status and *Varroa* occurrence

*Paenibacillus larvae* is suggested to be inactive in honeybee workers^24^ and is therefore not expected to have any direct effects on the bacterial community in the gut. The results of the AMOVA of the entire dataset are consistent with this expectation. However, we observed some effects on the microbiome when we performed RDA analysis (Fig. 2). Although we included varroosis as a factor in our analyses, the presence of *Varroa* in colonies did not have an additive effect on the microbiome in combination with *P. larvae*. This result corresponds to the experimental results of Alippi *et al*.^30^, who found that *Varroa* is not a vector for *P. larvae* ^30^. In bumblebees, defense against different pathogenic agents involves the participation of the bacterial community, which varies with infection^14–16^. Moreover, the honeybee microbiome changes under the pressure exerted by harmful parasites^17, 18^. Therefore, the apparent lack of a connection between *Varroa* occurrence and AFB in the present study was unexpected.

Interestingly, we identified *C. freundii* and *H. alvei* as synergic bacterial taxa to *P. larvae* in our study, whereas *E. faecalis*, *K. oxytoca*, *S. melliferum* and *M. morganii* were antagonistic within the community of pathogenic/environmental bacteria (Table 1). These findings raise the question of whether *C. freundii* and *H. alvei* are secondary bacterial invaders associated with AFB. Although little information is available regarding these bacteria, some authors have suggested that *C. freundii* and *H. alvei* are pathogenic to honeybees^31^. *Hafnia alvei* has also been sporadically described in the honeybee gut and is suggested to be an opportunistic pathogen that potentially interacts with other bacteria^32^. The correlations observed with *P. larvae* based on RDA analysis suggest that these bacteria occur in colonies that are weakened by AFB disease. The fact that *K. pneumoniae* and *M. plutonius* were more abundant in workers from asymptomatic colonies than in either control bees or colonies with AFB symptoms implies an association of these bacteria with the initial stage of AFB development in the colony. The decrease in *E. faecalis*, *K. oxytoca*, *S. melliferum* and *M. morganii* populations with the increase in *P. larvae* suggests possible negative influences of the pathogen on these bacteria. The negative correlations of the core bacteria *B. asteroides* and *L. mellis* with *P. larvae* (OTUs 8 and 9 in Fig. 2) similarly suggest negative influences of the pathogen. Because many active mechanisms by which bacteria influence other microbes have been described^33^, studies on these interactions remain a challenge for future work. It is possible that some metabolites of *P. larvae* influence the abundance of different bacteria in the honeybee colony. *Paenibacillus larvae* has been shown to produce siderophores^34^, which represents an important example of a competitive mechanism that involves a cooperative behavior^33^.

Finally, our results demonstrate the influence of AFB on the composition of the honeybee pupa microbiome. Our data obtained from pupae are preliminary, and more detailed examinations are warranted. The results suggest the prevalence of *L. apis*, *G. apicola*, *S. alvi*, and *F. perrara* in asymptomatic colonies in AFB-diseased apiaries, whereas in the asymptomatic, control colonies only *E. faecalis* was present. In the future, it will be of interest to study the underlying mechanism of this influence to explain these differences in bacterial presence and determine whether these findings are generally valid. It is not surprising that colonies with clinical signs of AFB were dominated by *P. larvae*, and in cases where pupae survive to adulthood, these individuals should be considered vectors of *P. larvae* in their colony.

### Methodological note – the cut-off microbiome

In this study, within the total microbiome, NGS was not an appropriate method to evaluate *P. larvae* in honeybee workers, but it was applicable for the analysis of the cut-off microbiome after the dominant core bacteria were reduced. This is because the sequences of symbiotic bacteria overlap with “rare” sequences of *P. larvae*. To enable data evaluation, we eliminated the core symbiotic bacteria from the microbiome; i.e., the sequences of core symbiotic bacteria were omitted from further analyses^15, 16^. Then, the pathogenic bacterium *P. larvae* was found to be prevalent in the bacterial community consisting of environmental/parasitic bacteria (Fig. 1). According to RDA analysis, *P. larvae* OTUs within the cut-off microbiome together with the presence of varroosis explained much of the variability in the bacterial microbiome and changes in the relative proportion of *P. larvae*, which were identifiable in both datasets (the total bacterial community and the community of pathogenic/environmental bacteria) (Figs 2 and 3).

## Materials and Methods

### Apiaries and sampling

Apiaries were selected according to their presence in the AFB zones denoted by the State Veterinary Administration of the Czech Republic; note that an AFB zone in Czechia is defined as a 5-km flight radius surrounding a diseased apiary. For the samples, we coded AFB as follows: (i) AFB0 (control): bees from outside the AFB zone with no signs of AFB; (ii) AFB1: bees from asymptomatic colonies within AFB apiaries; and (iii) AFB2: bees from colonies exhibiting clinical AFB symptoms. See Table 3 for a description of colonies, apiaries, sampling dates, AFB infestations and varroosis levels. After sampling, colonies exhibiting AFB symptoms in AFB apiaries were burned according to the regulations of Czechia; therefore, subsequent sample collection was impossible. Samples of the European honeybee, *A. mellifera carnica*, were collected from brood combs into polypropylene bags. Samples from each colony comprised three biological replicates of 10 worker bees, and we also sampled 10 pupae (purple eyes) from each colony where available. *Varroa* infestation levels were described according to Hubert *et al*.^18^ based on mites falling onto the bottom boards of a colony following treatment. The colonies were classified into three categories of varroosis infestation: level 1, 0–50 mites (low *Varroa* infestation; VDI1); level 2, 51–100 mites (moderate *Varroa* infestation; VDI2), and level 3, 101 or more mites (high *Varroa* infestation; VDI3).

**Table 3:** List of honeybee colonies and apiaries sampled from June to December 2014 with sample descriptions and environmental variables including geographical position, sampling time, AFB status and the presence of varroosis. We coded AFB as follows: (i) AFB0, control: bees from colonies outside the AFB zone with no signs of AFB; (ii) AFB1: bees from asymptomatic colonies in AFB apiaries; and (iii) AFB2: bees from colonies exhibiting clinical symptoms of AFB. Varroosis quantification was based on the number of mites on the bottom boards of a hive: level 1: 0–50 mites, level 2: 51–100 mites, and level 3: 101 mites or more.

Sample ID.	Geographical position			Sampling	Environmental factors		
	Site	N	E	date	Stage	AFB zone	Varroosis
H1AM1	Horni Lhota	49°35′43″	14°57′25″	26-Sep-2014	worker	2	2
H1AM2					worker		
H1AM3					worker		
H1AMK					pupae		
H1AMK1					pupae		
H1AMK2					pupae		
H1AMK3					pupae		
H2AM1	Horni Lhota	49°35′43″	14°57′25″	26-Sep-2014	worker	1	2
H2AM2					worker		
H2AM3					worker		
H2AMK					pupae		
H2AMK1					pupae		
R10AM1	Rataje	49°42′15″	14°58′14″	2-Oct-2014	worker	2	1
R10AM2					worker		
R10AM3					worker		
R10AMK					pupae		
R14AM1	Rataje	49°42′15″	14°58′14″	2-Oct-2014	worker	1	1
R14AM2					worker		
R14AM3					worker		
R14AMK					pupae		
S6AM1	Svrkyne-Hole	50°10′53″	14°15′49″	3-Dec-2014	worker	0	1
S6AM2				3-Jun-2014	worker		
S6AM3					worker		
S6AM4				29-Oct-2014	worker		
S6AM5					worker		
S6AM6					worker		
Z45AM1	Zdislavice	49°41′12″	14°58′28″	2-Oct-2014	worker	2	1
Z45AM2					worker		
Z45AM3					worker		
Z45AM4					worker		
Z45AMK					pupae		
Z51AM1	Zdislavice	49°41′12″	14°58′28″	2-Oct-2014	worker	1	1
Z51AM2					worker		
Z51AM3					worker		
Z51AMK					pupae		
Z706AM1	Stoky-Skrivanek	49°30′9″	15°35′19″	9-Jul-2014	worker	0	3
Z706AM2					worker		
Z706AM3					worker		
Z706AMK1					pupae		
Z706AMK2					pupae		

### DNA extraction from honeybees

Each worker sample was surface-sterilized by washing in pure ethanol, followed by rinsing three times with sterile phosphate-buffered saline (3.2 mM Na_2_HPO_4_, 0.5 mM KH_2_PO_4_, 1.3 mM KCl, and 135 mM NaCl) containing 0.05% w/w Tween® 20 detergent (PBS-T) (Sigma-Aldrich, Saint Louis, MO, USA) to remove the surface microflora. The pupae were not surface-treated. Worker or pupal samples were homogenized (whole-body homogenates) in 6 mL of sterile PBS-T in a glass Potter-Elvehjem homogenizer (Kavalier, Sazava, Czechia). The homogenates were transferred to sterile tubes (cat no. D1003; KRD, Prague, Czechia) and centrifuged (CL31R, Thermo Fisher Scientific, Waltham, MA, USA) at 845 × g for 5 minutes. The supernatants were mixed with 6 mL of phenol/chloroform/isopropanol (Roti-Phenol®, cat no. A156.2, Carl Roth, Karlsruhe, Germany) and centrifuged at 3,381 × g for 5 minutes. This step was repeated with the upper aqueous phase, which was extracted twice with chloroform:isopropanol (24:1) and centrifuged. The upper aqueous phase was then transferred into Eppendorf tubes and precipitated with a 0.1 volume of 3 M sodium acetate (cat no. S7899, Sigma-Aldrich, Saint Louis, MO, USA) and a 0.74 volume of isopropanol. For precipitation, the mixture was incubated at −20 °C for 20 minutes. Then, the tubes were centrifuged at 18,242 × g for 15 minutes, and the pellets were washed twice in 70% pure ethanol. The dried pellets were re-suspended in 200 µL of ddH_2_O (56 °C) via pipetting. Then, the DNA was cleaned using a Geneclean® Turbo kit (cat no. 1102–600, MP Biomedicals, Santa Ana, CA, USA). The DNA samples were stored at −40 °C until use.

### High-throughput sequencing (HTS)

The quality and presence of bacterial DNA in each sample was tested by performing PCR amplification using eubacterial primers, AFB primers and routinely used protocols^35, 36^. When amplicons were not obtained, the samples were replaced with new samples that were positive for amplicons. The DNA samples were sent to MR DNA for sequencing (http://mrdnalab.com, Shallowater, TX, USA). Sequencing of the V1–V3 portion of the 16 S rRNA gene was based on the 27Fmod and 519Rmod universal eubacterial primers on the Illumina MiSeq platform and the bTEFAP® process^37^. Read lengths were 300 bp, and forward and reverse reads were obtained. Sequences were processed as previously described^18^ using MOTHUR v.1.36.1 software^38^ according to the MiSeq standard operating procedure (MiSeq SOP^39^; http://www.mothur.org/wiki/MiSeq_SOP; accession date - 3/10/2016) and UPARSE with a USERCH pipeline^40^. The singletons and putative chimeras were discarded, and the OTUs were identified at 97% similarity according to the Ribosomal Database Project (http://rdp.cme.msu.edu) using training set no. 15^41^. Then, the representative sequences were processed using the BLASTn program on the NCBI platform (https://blast.ncbi.nlm.nih.gov/)^42^, and OTUs similar to chloroplasts and Archaea were removed. The best search hits were chosen based on the highest bit scores. Abundance data were then reincorporated into the dataset by mapping the initial sequences against the representative OTUs. The data were deposited in GenBank as SRA project no. SRP093442, and a list of samples is provided in Table S4. The taxonomic features of the samples were visualized by performing KRONA projections^43^, and abundance data were transformed into a shared file and processed in MOTHUR.

### Data analyses

Based on the NCBI and RDP matches, we identified honeybee symbionts and pathogenic/environmental bacteria^44^ and constructed a shared file for all OTUs and a separate shared file for pathogenic/environmental OTUs according to the analyses of Cariveau *et al*.^15, 16^. The OTU classifications are presented in Tables S1 and S2. Both shared files were standardized by subsampling for a minimal number of sequences in sample, i.e., 14,741 sequences for all bacteria and 104 sequences for pathogenic/environmental bacteria in MOTHUR.

Alpha diversity was assessed by calculating the inverse Simpson index and the number of OTUs, and the effects of the factor AFB status were tested by performing the nonparametric Kruskal–Wallis test using XLSTAT software (http://www.xlstat.com/en/, Addinsoft, New York, NY, USA).

Beta diversity was studied by performing distance-based RDA (db-RDA), sometimes called the constrained analysis of principal coordinates. A Bray-Curtis dissimilarity matrix was used as the basis for this examination^45^. The main purpose was to determine if the factor of AFB zone substantially influenced only the pathogenic OTUs or all of the OTUs. Therefore, as with the AMOVA and HOMOVA analyses, two distinct OTU subsamples were used separately. In addition to the effects of AFB status, the effects of *Varroa* infestation level (factor with three levels) (Table 3) were explored. During the construction of db-RDA models, covariates such as geographic coordinates (longitude and latitude) and the time of bee sample collection were included, and VIF values^46^ were controlled for when adding environmental explanatory variables to the models. Moreover, the decision to include variables in the models was supported by the forward selection implemented in the “packfor” R package^47^ or permutational ANOVA-like tests performed with the same statistical package, the “vegan” R package^48^, with which the db-RDA models themselves were constructed. The partial db-RDA models were of particular interest in that they suppressed the potential risk of multicollinearity when regressing the response variables on the matrix composed of environmental variables. Additionally, the significance of canonical axes, the result of the PCA stage of the RDA, was determined by means of the permutational tests regardless of the proportion of variability that they explained. Regardless of their significance, the first two axes formed the basis of the correlation triplots in which site (sample) scores were expressed as the weighted sums of species given the dissimilarity matrix. Population-level analyses were carried out by comparing AFB zones and microbiomes for both the total bacteria and pathogenic/environmental bacteria datasets with METASTATS^49^ using 100,000 permutations in MOTHUR. To allow the results to be visualized more clearly, we have multiplied the vectors of the environmental variables AFB and varr2 2-fold and the OTU vectors 4-fold in the triplot visualizations presented in Figs 2 and 3.

## Electronic supplementary material

Supplementary Tables S1–S4

Supplementary Figure S1

## References

[1] OIE. Chapter 9.2. Infection of honey bees with *Paenibacillus larvae* (American foulbrood). In: *OIE Terrestrial Animal Health Code, vol. 2* (OIE - World Organisation for Animal Health) http://www.oie.int/index.php?id=169&L=0&htmfile=chapitre_paenibacillus_larvae.htm (2016).

[2] Ash, C., Priest, F. G. & Collins, M. D. Molecular identification of rRNA group 3 bacilli (Ash, Farrow, Wallbanks and Collins) using a PCR probe test: proposal for the creation of a new genus *Paenibacillus*. *Antonie Van Leeuwenhoek* **64**, 253–260 (1993/1994). 10.1007/BF00873085 8085788

[3] White, G. F. *The bacteria of the apiary, with special reference to bee diseases*. (Bureau of Entomology, U. S. Department of Agriculture, 1906).

[4] Genersch E (2010). American foulbrood in honeybees and its causative agent. Paenibacillus larvae. J. Invertebr. Pathol..

[5] Hansen H, Brodsgaard CJ (1999). Foulbrood diseases. Apiacta.

[6] Lindstrom A, Fries I (2005). Sampling of adult bees for detection of American foulbrood (*Paenibacillus larvae* subsp. *larvae*) spores in honey bee (*Apis mellifera*) colonies. J. Apic. Res.

[7] Gillard M, Charriere JD, Belloy L (2008). Distribution of *Paenibacillus larvae* spores inside honey bee colonies and its relevance for diagnosis. J. Invertebr. Pathol..

[8] Garrido-Bailon E (2013). The prevalence of the honeybee brood pathogens *Ascosphaera apis*, *Paenibacillus larvae* and *Melissococcus plutonius* in Spanish apiaries determined with a new multiplex PCR assay. Microb. Biotechnol.

[9] Lindstrom A (2008). Distribution of *Paenibacillus larvae* spores among adult honey bees (*Apis mellifera*) and the relationship with clinical symptoms of American foulbrood. Microb. Ecol..

[10] de Graaf DC (2013). Standard methods for American foulbrood research. J. Apic. Res.

[11] Machova M (1993). Resistance of *Bacillus larvae* in beeswax. Apidologie.

[12] Gochnauer TA, Corner J (1974). Detection and identification of *Bacillus larvae* in a commercial sample of bee-collected pollen. J. Apic. Res.

[13] Moran NA (2015). Genomics of the honey bee microbiome. Curr. Opin. Insect Sci..

[14] Koch H, Schmid-Hempel P (2011). Socially transmitted gut microbiota protect bumble bees against an intestinal parasite. Proc. Natl. Acad. Sci. USA.

[15] Cariveau DP, Powell JE, Koch H, Winfree R, Moran NA (2014). Variation in gut microbial communities and its association with pathogen infection in wild bumble bees (*Bombus*). ISME J..

[16] Cariveau DP, Powell JE, Koch H, Winfree R, Moran NA (2014). Erratum to “Variation in gut microbial communities and its association with pathogen infection in wild bumble bees (*Bombus*) [ISME J. 8, 2369–2379 (2014)]”. ISME J..

[17] Hubert J (2017). Changes in the bacteriome of honey bees associated with the parasite *Varroa destructor*, and pathogens *Nosema* and *Lotmaria passim*. Microb. Ecol..

[18] Hubert J (2016). Comparison of *Varroa destructor* and worker honeybee microbiota within hives indicates shared bacteria. Microb. Ecol..

[19] Erban, T. *et al*. Bacterial community associated with honeybees *Apis mellifera* affected by European foulbrood. *PeerJ* (in review) (2017). 10.7717/peerj.3816 PMC5619233 28966892

[20] Lindstrom A, Korpela S, Fries I (2008). The distribution of *Paenibacillus larvae* spores in adult bees and honey and larval mortality, following the addition of American foulbrood diseased brood or spore-contaminated honey in honey bee (*Apis mellifera*) colonies. J. Invertebr. Pathol..

[21] Gende L (2011). Searching for an American foulbrood early detection threshold by the determination of *Paenibacillus larvae* spore load in worker honey bees. Bull. Insectol.

[22] Fries I, Lindstrom A, Korpela S (2006). Vertical transmission of American foulbrood (*Paenibacillus larvae*) in honey bees (*Apis mellifera*). Vet. Microbiol..

[23] Peters M, Kilwinski J, Beringhoff A, Reckling D, Genersch E (2006). American foulbrood of the honey bee: occurrence and distribution of different genotypes of *Paenibacillus larvae* in the administrative district of Arnsberg (North Rhine-Westphalia). J. Vet. Med. B.

[24] Riessberger-Galle U, von der Ohe W, Crailsheim K (2001). Adult honeybee’s resistance against *Paenibacillus larvae larvae*, the causative agent of the American foulbrood. J. Invertebr. Pathol..

[25] Mill AC (2014). Clustering, persistence and control of a pollinator brood disease: epidemiology of American foulbrood. Environ. Microbiol..

[26] Pfeiffer KJ, Crailsheim K (1998). Drifting of honeybees. Insect. Soc.

[27] Shimanuki H, Knox DA (1997). Bee health and international trade. Rev. sci. tech..

[28] Morrissey BJ (2015). Biogeography of *Paenibacillus larvae*, the causative agent of American foulbrood, using a new multilocus sequence typing scheme. Environ. Microbiol..

[29] Hansen H, Rasmussen B (1986). The investigation of honey from bee colonies for *Bacillus larvae*. Dan. J. Plant Soil Sci..

[30] Alippi AM, Albo GN, Marcangeli J, Leniz D, Noriega A (1995). The mite *Varroa jacobsoni* does not transmit American foulbrood from infected to healthy colonies. Exp. Appl. Acarol..

[31] Lyapunov YE, Kuzyaev RZ, Khismatullin RG, Bezgodova OA (2008). Intestinal enterobacteria of the hibernating *Apis mellifera mellifera* L. bees. Microbiology.

[32] Tian B, Moran NA (2016). Genome sequence of *Hafnia alvei* bta3_1, a bacterium with antimicrobial properties isolated from honey bee gut. Genome Announc.

[33] Hibbing ME, Fuqua C, Parsek MR, Peterson SB (2010). Bacterial competition: surviving and thriving in the microbial jungle. Nat. Rev. Microbiol..

[34] Hertlein G (2014). Production of the catechol type siderophore bacillibactin by the honey bee pathogen *Paenibacillus larvae*. PLoS One.

[35] Lane, D. J. 16S/23S rRNA sequencing. In: eds. Stackebrandt, E. & Goodfellow, M. *Nucleic acid techniques in bacterial systematics*. (John Wiley and Sons, 1991), p. 115–175.

[36] Dobbelaere W, de Graaf DC, Peeters JE (2001). Development of a fast and reliable diagnostic method for American foulbrood disease (*Paenibacillus larvae* subsp. *larvae*) using a 16S rRNA gene based PCR. Apidologie.

[37] Chiodini RJ (2015). Microbial population differentials between mucosal and submucosal intestinal tissues in advanced Crohn’s disease of the ileum. PLoS One.

[38] Schloss PD (2009). Introducing mothur: open-source, platform-independent, community-supported software for describing and comparing microbial communities. Appl. Environ. Microbiol..

[39] Kozich JJ, Westcott SL, Baxter NT, Highlander SK, Schloss PD (2013). Development of a dual-index sequencing strategy and curation pipeline for analyzing amplicon sequence data on the MiSeq Illumina sequencing platform. Appl. Environ. Microbiol..

[40] Edgar RC (2013). UPARSE: highly accurate OTU sequences from microbial amplicon reads. Nat. Methods.

[41] Cole JR (2014). Ribosomal Database Project: data and tools for high throughput rRNA analysis. Nucleic Acids Res.

[42] Altschul SF (1997). Gapped BLAST and PSI-BLAST: a new generation of protein database search programs. Nucleic Acids Res.

[43] Ondov BD, Bergman NH, Phillippy AM (2011). Interactive metagenomic visualization in a Web browser. BMC Bioinformatics.

[44] Engel P (2016). The bee microbiome: impact on bee health and model for evolution and ecology of host-microbe interactions. mBio.

[45] Engel P (2013). Standard methods for research on *Apis mellifera* gut symbionts. J. Apic. Res..

[46] Kutner, M. H., Nachtsheim, C. J., Neter, J. & Li, W. *Applied linear statistical models*, *5*^*th*^*edn*. (McGraw-Hill Irwin, 2005).

[47] Dray, S., Legendre, P. & Blanchet, G. packfor: Forward selection with permutation (Canoco p. 46). *R-Forge, The R Project for Statistical Computing* http://R-Forge.R-project.org/projects/sedar/ (2013).

[48] Oksanen, J. *et al*. vegan: Community Ecology Package. *CRAN - The Comprehensive R Archive Network* http://CRAN.R-project.org/package=vegan (2016).

[49] White JR, Nagarajan N, Pop M (2009). Statistical methods for detecting differentially abundant features in clinical metagenomic samples. PLoS Comput. Biol..

