# Supplementary material for: Honeybee (Apis mellifera)-associated bacterial community affected by American foulbrood: detection of Paenibacillus larvae via microbiome analysis
Source: Sci Rep. 2017 Jul 11;7:5084. doi: 10.1038/s41598-017-05076-8 (PMC5506040; doi:10.1038/s41598-017-05076-8)

Supplementary Information

Figure S1

Journal: Scientific Reports

Honeybee (*Apis mellifera*)-associated bacterial community affected by American foulbrood: detection of *Paenibacillus larvae* via microbiome analysis

Tomas Erban[1], Ondrej Ledvinka[2], Martin Kamler[3], Marta Nesvorna[1], Bronislava Hortova[1], Jan Tyl[3], Dalibor Titera[3,4], Martin Markovic[1] & Jan Hubert[1]

[1]Crop Research Institute, Drnovska 507/73, Prague 6-Ruzyne, CZ-161 06, Czechia

[2]Czech Hydrometeorological Institute, Na Sabatce 2050/17, Prague 412, CZ-143 06, Czechia

[3]Bee Research Institute at Dol, Maslovice-Dol 94, Libcice nad Vltavou, CZ-252 66, Czechia

[4]Department of Zoology and Fisheries, Faculty of Agrobiology, Food and Natural Resources, Czech University of Life Sciences Prague, Prague 6-Suchdol, Czechia

***Corresponding author:**

Tomas Erban

Crop Research Institute

Proteomics and Metabolomics Laboratory

Drnovska 507/73, Prague 6-Ruzyne

CZ-16106

Czechia

E-mail: arachnid@centrum.cz

Figure S1. Krona projections of bacteriome of pupae of *Apis mellifera* in colonies with (**AFB2**) and without (**AFB1**) clinical signs of AFB, and control (**AFB0**) samples.

AFB2 – site: Horni Lhota

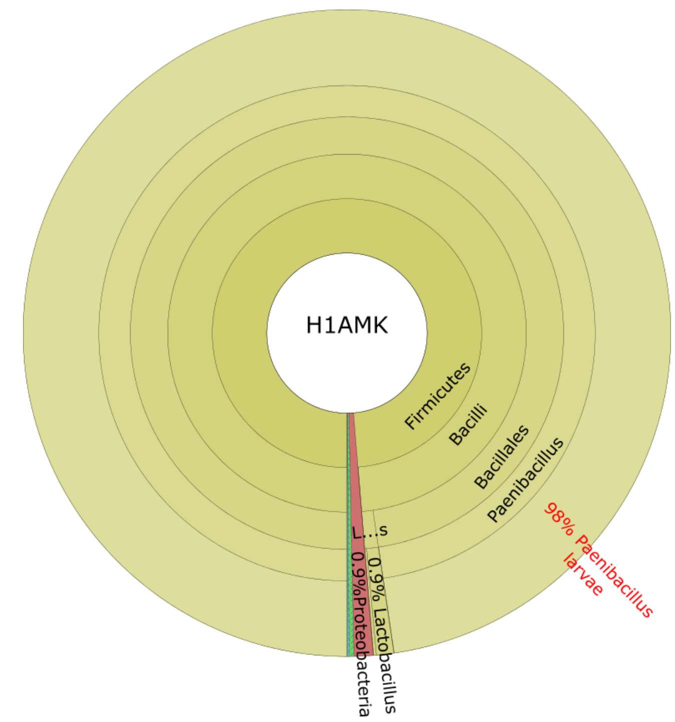

AFB2 – site: Horni Lhota

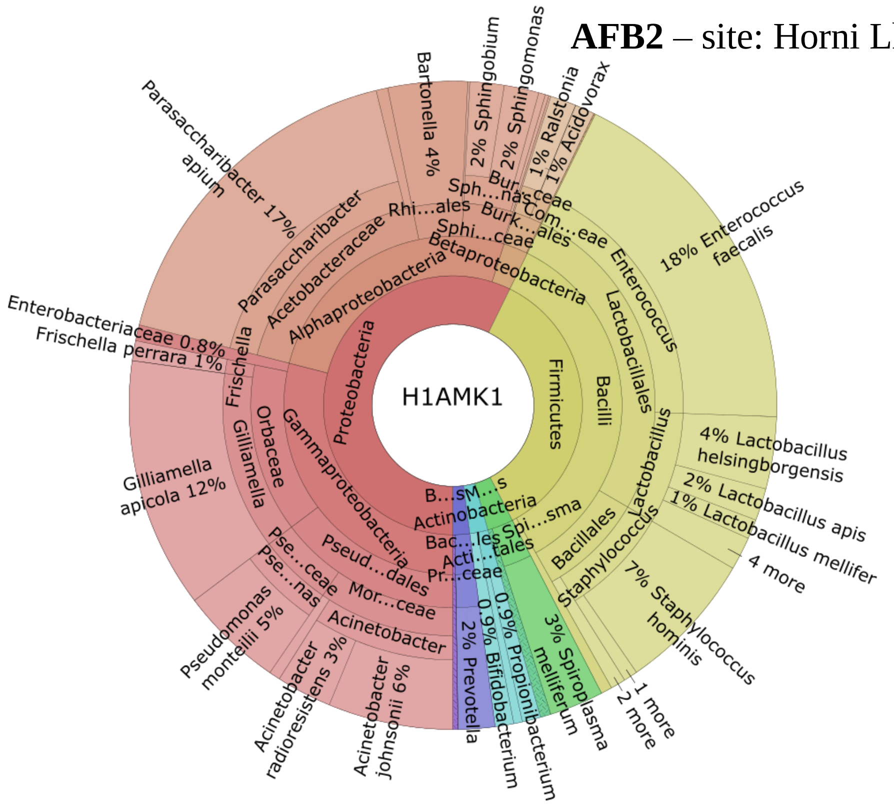

AFB2 – site: Horni Lhota

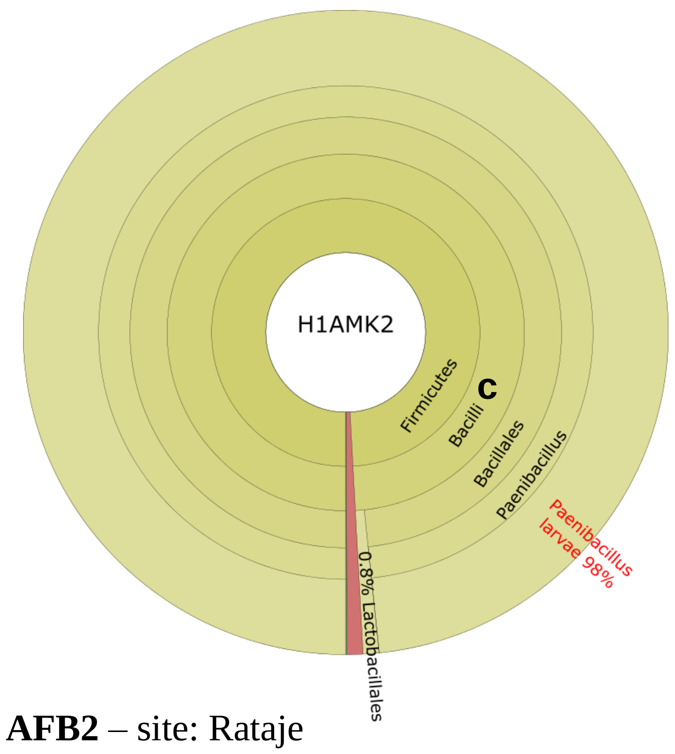

AFB2 – site: Horni Lhota

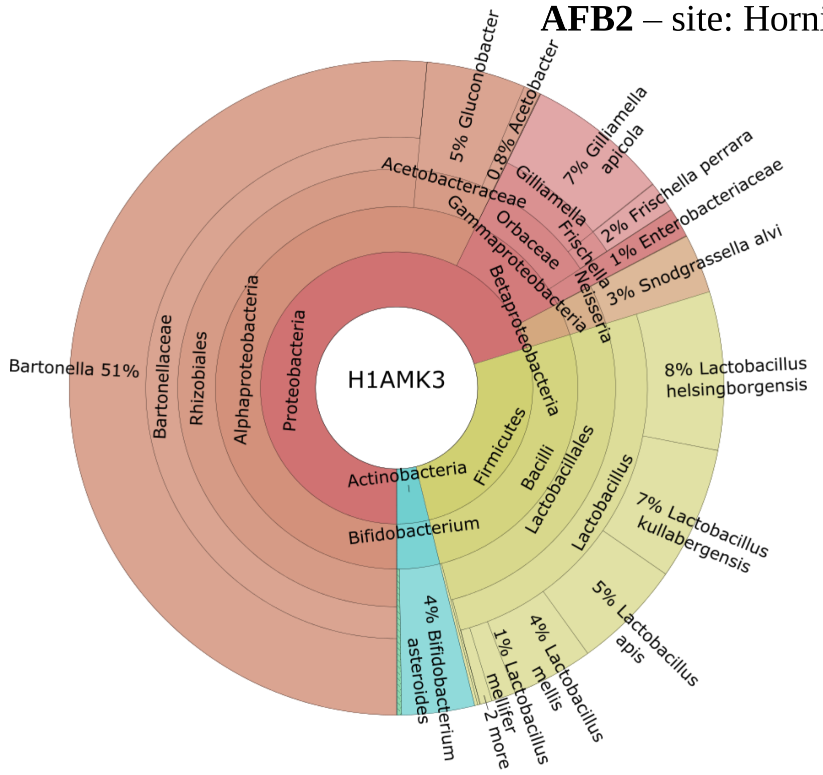

AFB2 – site: Rataje

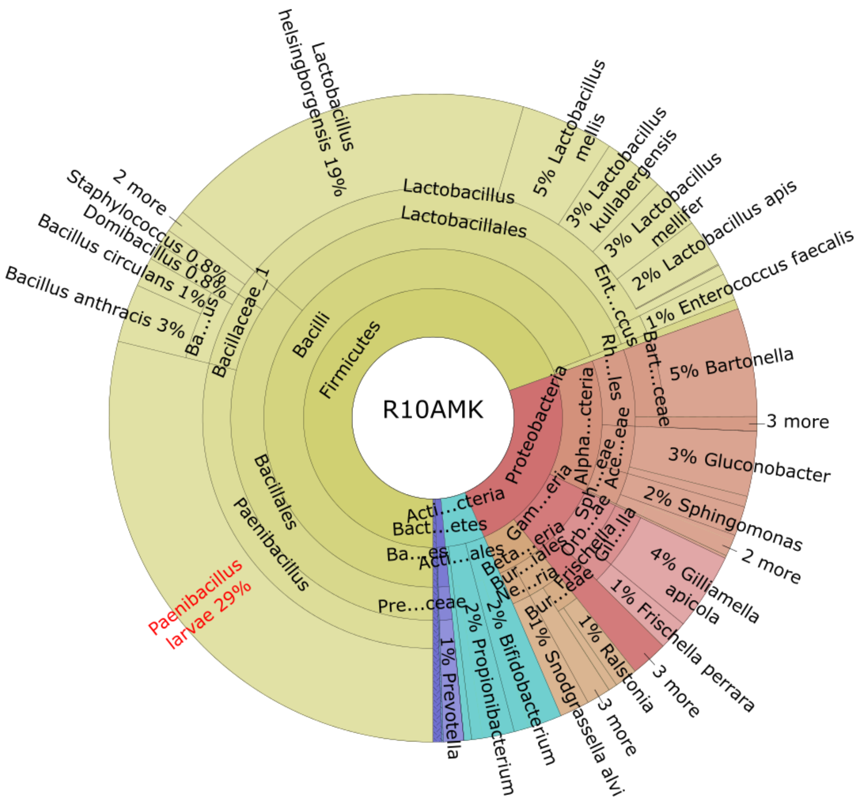

AFB2 – site: Zdislavice

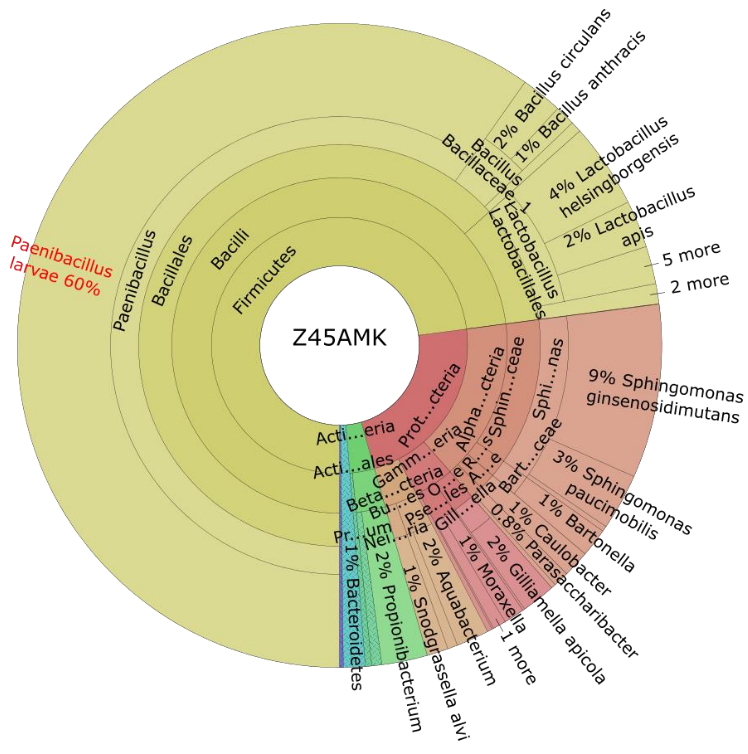

Figure S1 continuing. Krona projections of bacteriome of pupae of *Apis mellifera* in colonies with (**AFB2**) and without (**AFB1**) clinical signs of AFB, and control (**AFB0**) samples

AFB1 – site: Horni Lhota

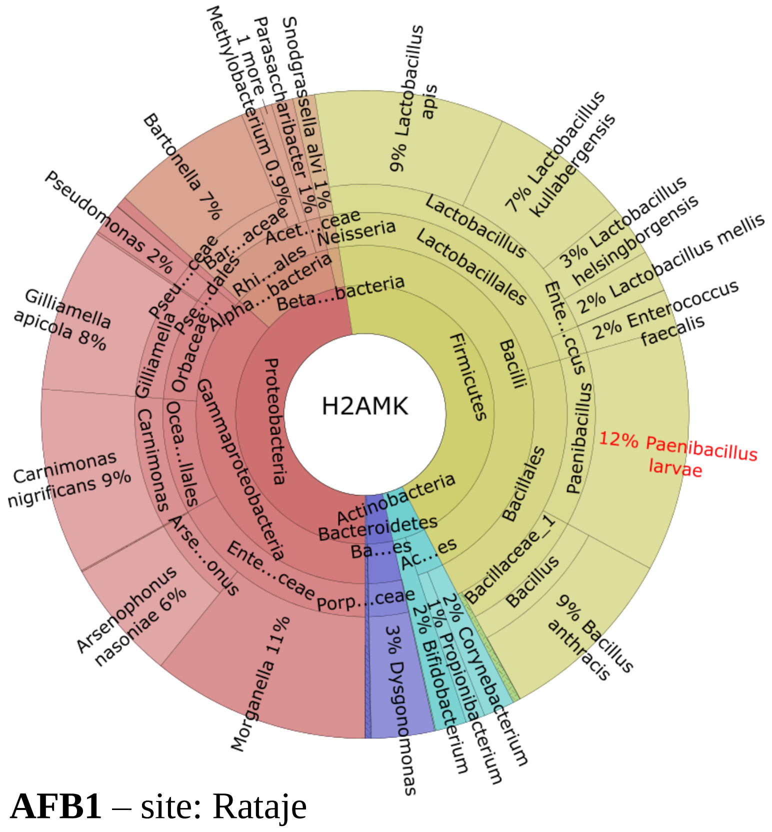

AFB1 – site: Horni Lhota

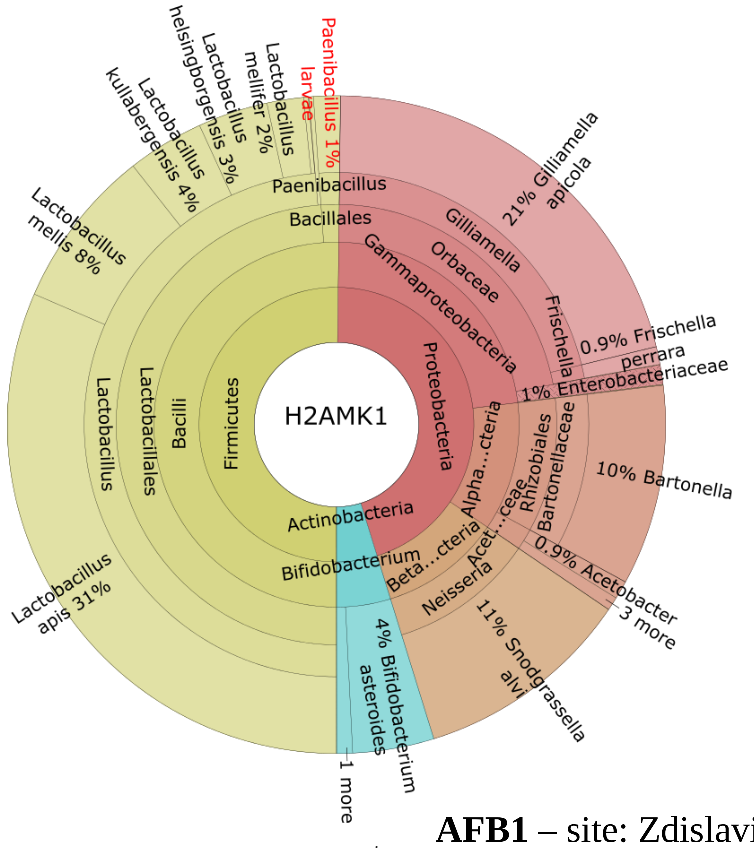

AFB1 – site: Rataje

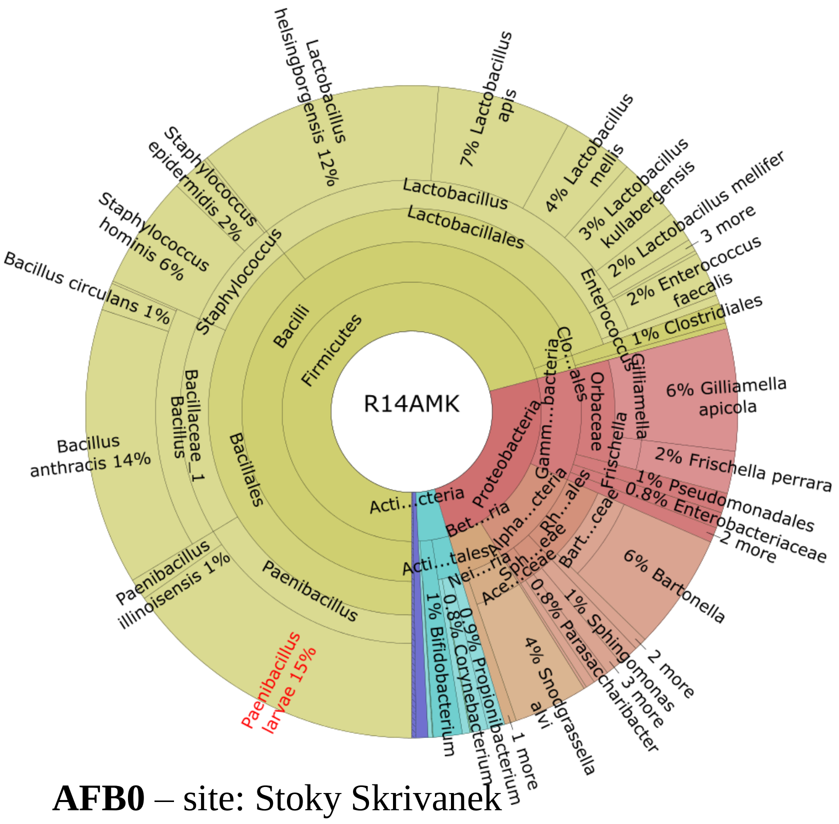

AFB1 – site: Zdislavice

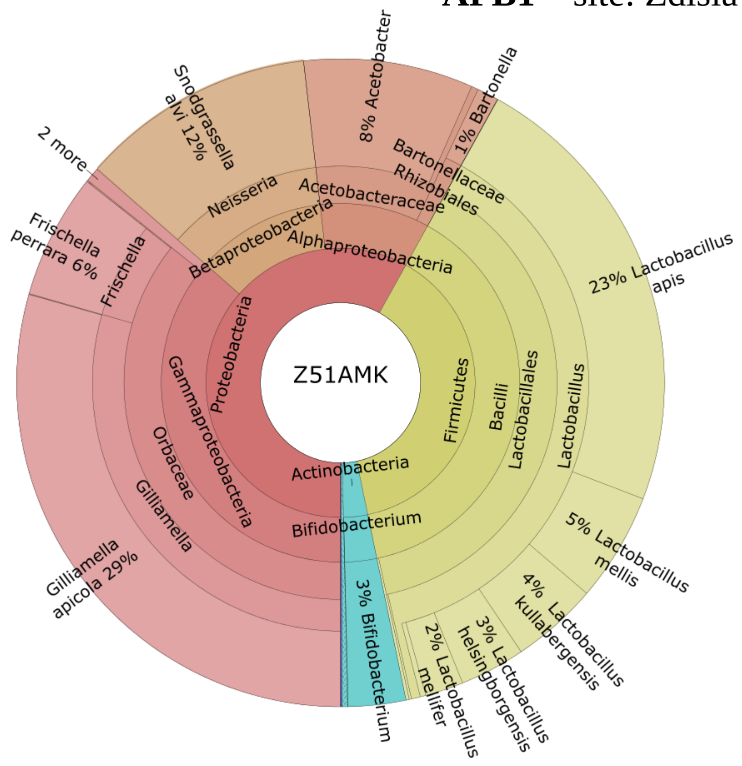

AFB0 – site: Stoky Skrivanek

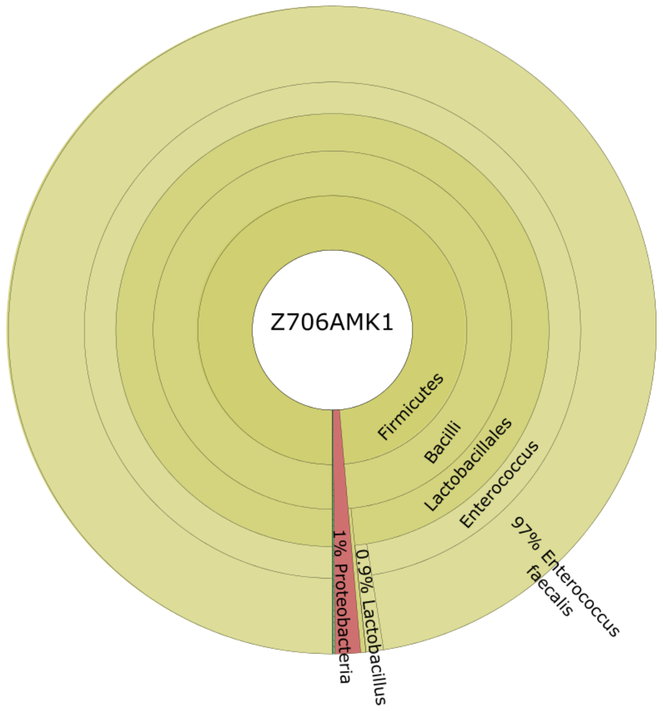

AFB0 – site: Stoky Skrivanek

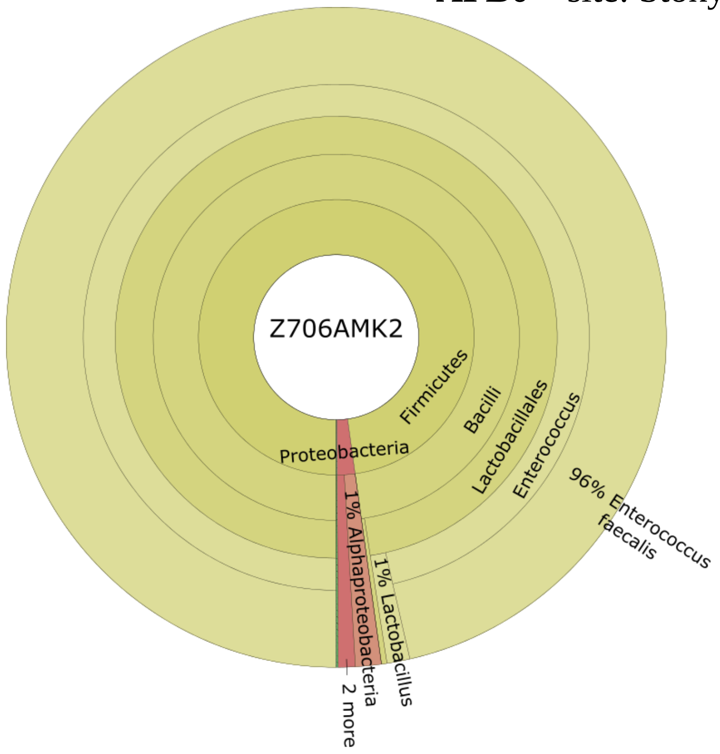

Supplement: Supplementary file 2 — Supplementary Figure S1 [file 41598_2017_5076_MOESM2_ESM.pdf]
